# Inflammation, glucose, and vascular cell damage: the role of the pentose phosphate pathway

**DOI:** 10.1186/s12933-016-0397-2

**Published:** 2016-06-01

**Authors:** Concepción Peiró, Tania Romacho, Verónica Azcutia, Laura Villalobos, Emilio Fernández, Juan P. Bolaños, Salvador Moncada, Carlos F. Sánchez-Ferrer

**Affiliations:** Departamento de Farmacología, Facultad de Medicina, Universidad Autónoma de Madrid, 29029 Madrid, Spain; Instituto de Biología Funcional y Genómica, Universidad de Salamanca-CSIC, 37007 Salamanca, Spain; Wolfson Institute for Biomedical Research, University College London, London, WC1E 6BT UK; Paul Langerhans-Group, Integrative Physiology, German Diabetes Center, Auf’m Hennekamp 65, 40225 Düsseldorf, Germany; Department of Pathology, University of Michigan, Ann Arbor, MI 48109 USA; Institute of Cancer Sciences, Manchester Cancer Research Centre, University of Manchester, Wilmslow Road, Manchester, M20 4QL UK

**Keywords:** Vascular cells, High glucose, Inflammation, Pentose phosphate pathway, Oxidative stress

## Abstract

**Background:**

Hyperglycemia is acknowledged as a pro-inflammatory condition and a major cause of vascular damage. Nevertheless, we have previously described that high glucose only promotes inflammation in human vascular cells previously primed with pro-inflammatory stimuli, such as the cytokine interleukin (IL)1β. Here, we aimed to identify the cellular mechanisms by which high glucose exacerbates the vascular inflammation induced by IL1β.

**Methods:**

Cultured human aortic smooth muscle cells (HASMC) and isolated rat mesenteric microvessels were treated with IL1β in medium containing 5.5–22 mmol/L glucose. Glucose uptake and consumption, lactate production, GLUT1 levels, NADPH oxidase activity and inflammatory signalling (nuclear factor-κB activation and inducible nitric oxide synthase expression) were measured in HASMC, while endothelium-dependent relaxations to acetylcholine were determined in rat microvessels. Pharmacological inhibition of IL1 receptors, NADPH oxidase and glucose-6-phosphate dehydrogenase (G6PD), as well as silencing of G6PD, were also performed. Moreover, the pentose phosphate pathway (PPP) activity and the levels of reduced glutathione were determined.

**Results:**

We found that excess glucose uptake in HASMC cultured in 22 mM glucose only occurred following activation with IL1β. However, the simple entry of glucose was not enough to be deleterious since over-expression of the glucose transporter GLUT1 or increased glucose uptake following inhibition of mitochondrial respiration by sodium azide was not sufficient to trigger inflammatory mechanisms. In fact, besides allowing glucose entry, IL1β activated the PPP, thus permitting some of the excess glucose to be metabolized via this route. This in turn led to an over-activation NADPH oxidase, resulting in increased generation of free radicals and the subsequent downstream pro-inflammatory signalling. Moreover, in rat mesenteric microvessels high glucose incubation enhanced the endothelial dysfunction induced by IL1β by a mechanism which was abrogated by the inhibition of the PPP.

**Conclusions:**

A pro-inflammatory stimulus like IL1β transforms excess glucose into a vascular deleterious agent by causing an increase in glucose uptake and its subsequent diversion into the PPP, promoting the pro-oxidant conditions required for the exacerbation of pro-oxidant and pro-inflammatory pathways. We propose that over-activation of the PPP is a crucial mechanism for the vascular damage associated to hyperglycemia.

**Electronic supplementary material:**

The online version of this article (doi:10.1186/s12933-016-0397-2) contains supplementary material, which is available to authorized users.

## Background

At present, hyperglycaemia is recognized as an independent risk factor for cardiovascular disease. Thus, it has been proposed that high glucose per se induces oxidative stress that results in vascular damage, due to an excess entry of glucose into the cell leading to the over-production of superoxide anions in the mitochondria [1]. However, clinical trials have shown that strict control of glycaemia leads only to a modest reduction in macrovascular diabetic complications [2]. Thus, the link between hyperglycaemia and atherosclerosis is still not fully understood [3], while the role for mitochondrial superoxide as the culprit of diabetes complications is now questioned [4, 5].

We have previously shown that exposure to high concentrations of glucose does not cause inflammation in human vascular cells unless the cells are primed with an inflammatory stimulus such as interleukin (IL)1β or tumour necrosis factor (TNF)α [6, 7]. This led us to conclude that a background inflammatory condition is necessary for the elevation of extracellular glucose to become deleterious in the vasculature, which can explain why glycaemic control alone is not sufficient to avoid diabetic vascular damage. Understanding the mechanisms by which an inflammatory environment transforms high glucose into a deleterious agent can provide new therapeutic targets for preventing diabetic complications.

In the present work, we have explored in human vascular smooth muscle cells the role of the pro-inflammatory cytokine IL1β on the uptake and utilization of glucose and the possible mechanisms linking glucose metabolism with pro-inflammatory signalling. Furthermore, using rat mesenteric microvessels, we investigated whether how endothelial dysfunction induced by IL1β is potentiated by high glucose concentrations. We propose that an excess of glucose entry is diverted through the pentose phosphate pathway (PPP), providing additional substrate for the enzyme NADPH oxidase and results in a pro-oxidant environment that exacerbates inflammation.

## Methods

### Ethical approval

The Ethics Committees for Clinical Research from Hospital Universitario de Getafe (reference numbers 11-20 and 14-117) and Hospital Universitario La Paz (reference numbers PI-1111 and PI-1878) approved the protocol in human cell cultures. The studies on animals were performed according to national and European guidelines (2010/63/EU), approved by the ethics committee of Universidad Autónoma de Madrid (CEI 27-670), and developed in registered animal facilities (ES-28079-000097).

### Cell culture

Human aortic smooth muscle cells (HASMC) were obtained by enzymatic dissociation from the aortae of five organ donors, who had no evidences for cardiometabolic diseases or risk factors, and according to Spanish legal regulations as previously described [6]. The cells were routinely cultured in Dulbecco’s Modified Eagle Medium (DMEM, Biological Industries, Beit-Hamek, Israel) supplemented with 10 % fetal calf serum (FCS, Biological Industries), 100 µg/ml streptomycin (Reig-Jofré, Barcelona, Spain), 100 U/ml penicillin (ERN SA, Barcelona, Spain) and 2.5 µg/ml amphotericin B (Sigma Chemical Co., St. Louis, MO, USA). At confluence, the cells were passaged using a 0.02 % EDTA-0.05 % trypsin solution (Biological Industries). The cells were counted using a hemocytometer. Cell characterization was carried out based on both cell morphology and indirect immunofluorescence staining of α-smooth muscle actin. For experiments, confluent cells were serum-deprived for 24 h prior to the addition of the different test compounds in serum-free DMEM supplemented with 0.1 % bovine serum albumin (BSA, Sigma). Confluent cultures between passages 3 and 12 were used.

### Glucose consumption and lactate production

The cells were plated onto 48-well plates at a density of 20,000 cells per well. After serum-deprivation the cells were incubated for 24 h in DMEM with 0.1 % BSA and different initial concentrations of glucose (5.5, 11, or 22 mmol/L); in some experiments, the cultures were also treated with 1–10 ng/mL interleukin-1β (IL1β; Peprotech, London, UK) or 0.5 mmo/L sodium azide (Sigma). Glucose consumption and lactate release over 8 or 24 h of incubation were determined by measuring the respective levels in the culture medium at time 0 and after 8 or 24 h using commercial kits (Glucose HK Assay kit, Sigma; Lactate Reagent kit, Trinity Biotech, Wicklow, Ireland). Glucose consumption was calculated as the difference between initial and final glucose levels, while lactate production was calculated as the difference between final and initial lactate levels. Results were normalized by cell number. Both glucose consumption and lactate production were expressed as pmol per cell over 8 or 24 h.

### Cytochalasin B-sensitive ^3^H-deoxyglucose uptake

Cytochalasin B-sensitive ^3^H-deoxyglucose uptake was used as an indicator of the cellular GLUT1 transporter capacity. Briefly, cells were incubated for 18 h in 24-well plates containing DMEM with 0.1 % BSA and 5.5 or 22 mmol/L glucose; in some experiments, the cells were also treated with 1–10 ng/mL IL1β 1 ng/mL anakinra (Biovitrum, SOBI, Stockholm, Sweden), or 0.5 mmol/L sodium azide. The 18 h optimal period for such treatments was established in preliminary experiments (data not shown). After washing in a Krebs–Ringer solution free of glucose (KRS; composition in mmol/L, NaCl 136, KCl 4.7, MgSO_4_ 1.25, CaCl_2_ 1.25, pH 7.4 HEPES 10), the cultures were pre-incubated for 15 min in KRS without d-glucose, in the presence or absence of the GLUT1 transporter blocker cytochalasin B (Sigma; 20 µmol/L). The cultures were then incubated for 5 min in KRS without cold d-glucose containing 1 µCi/mL ^3^H-deoxyglucose (activity 8.00 Ci mmol; Amersham, Buckinghamshire, UK), in the absence or presence of cytochalasin B. The optimal 5 min time for such incubation period was established in preliminary experiments (data not shown). After washing with KRS, the protein content was determined by the bicinchoninic acid (BCA) assay and the radioactivity incorporated to the cells was measured in a liquid scintillation counter (Tri-Carb 2800 TR, Perkin Elmer, Waltham, MA, USA). Results were expressed in dpm/µg protein.

### GLUT1 lentiviral infection

A pCB6 plasmid containing the human GLUT1 cDNA sequence was a kind gift from Prof. David James (Garvan Institute of Medical Research, Darlinghurst Sydney NSW, Australia). The GLUT1 cDNA was subcloned into a lentiviral vector (pCDH-CMV-MCS-EF1-Puro, System Biosciences SBI) using the XbaI restriction site. All plasmids were confirmed by PCR screening and DNA sequencing. Viral particles were generated according to the manufacturer’s protocol (pPACKH1 Lentivector Packaging Kit, SBI, Mountain View, CA, USA). The cells were infected with viral particles (GLUT1+) in the presence of 4 µg/mL polybrene (Sigma) and selected in 1 µg/mL puromycin (Sigma) for 3 days. Matched cells subjected to infection with empty vector lentiviral plasmids (EV) were used as controls.

### Rt-pcr

For GLUT1 mRNA quantification, total RNA was extracted from vascular cells after 18 h of treatment using a commercial kit (RNeasy, Qiagen; Crawley, UK) and quantified by absorbance at 260 nm. Aliquots of 1 µg RNA were reverse-transcribed using Moloney murine leukemia virus-reverse transcriptase (Sigma). The resulting cDNA was amplified using a human GLUT1 gene-specific relative RT-PCR kit (Ambion, Austin, TX, USA) containing an 18S internal standard, according to the manufacturer’s instructions. The reaction was conducted in a Peltier PTC100 thermocycler (M&J Research, Waltham, MA, USA) with an initial denaturation step at 95 °C for 3 min, followed by 23 cycles each consisting of incubation at 94 °C for 30 s, 59 °C for 30 s, and 72 °C for 30 s. Aliquots of the resulting PCR were loaded on 2 % agarose gels containing ethidium bromide, the resulting bands were visualized under ultraviolet light and quantified using NIH Image free software.

### Indirect immunofluorescence

As previously described [6, 7], cells grown on glass coverslips were exposed for 18 h to the different treatments, fixed with acetone at −20 °C and blocked in a PBS solution containing 1 % BSA and 0.1 % Triton X-100. The cells were then incubated with a primary polyclonal antibody against GLUT1 (dilution 1/50; Neomarkers, Fremont, CA, USA), followed by incubation with an appropriate Alexa Fluor 488-secondary antibody (dilution 1/200; Molecular Probes-Invitrogen Corporation). Nuclei were counterstained with 4′,6-diamino-2-phenylindole (DAPI). Cells were observed with an Eclipse TE300 epifluorescence microscope (Nikon, Tokyo, Japan) and a spectral confocal microscope (Leica TCSSP5-AOBS, Leica microsystems, Heidelberg, GMBH, Germany). Confocal images were analyzed with LAS AF software, version 1.5.1 Build 869 (Leica).

### Western blotting

After washing with PBS, the cells were subjected to extraction in lysis buffer containing 10 mmol/L Tris pH 7.4, 1 % sodium dodecyl sulfate (SDS), 10 mmol/L sodium orthovanadate, 2 mmol/L phenylmethylsulfonyl fluoride and 12.5 µg/mL aprotinin (all from Sigma). Proteins (20 µg per lane) were equally loaded and separated on 8 % SDS-PAGE and transferred onto a nitrocellulose membrane (BioRad Laboratories, Madrid, Spain). After blocking overnight, the membrane was incubated for 1 h at room temperature with polyclonal antibodies against iNOS (dilution 1/10,000; Transduction Laboratories, Lexington, KY, USA) or glucose-6-phosphate dehydrogenase (G6PD, dilution 1/5000, Sigma), followed by incubation for 1 h with a horseradish peroxidase-conjugated secondary antibody (dilution 1/10,000; Chemicon, Temecula, CA, USA), as described previously [6]. Immunoreactive bands were detected using an enhanced chemiluminescence detection kit (GE Healthcare, Uppsala, Sweden) and quantified by densitometry using NIH Image software. The membranes were stripped and reprobed with an anti α-tubulin primary antibody (dilution 1/10,000; Sigma) to ensure equal loading.

### Nuclear extracts and electrophoretic mobility shift assay (EMSA)

After 18 h of treatment, nuclear extracts from cell cultures were prepared as previously described [6, 7]. For binding reactions, nuclear extracts (5 μg) were incubated on ice for 15 min in a reaction buffer (pH 7.0 40 mmol/L HEPES, 140 mmol/L NaCl, 5 mmol/L dithiothreitol, 10 μg/mL BSA, 0.01 % Nonidet P-40, 4 % Ficoll and 0.05 μg/mL poly [dI-dC]poly [dI-dC]). A commercial oligonucleotide (Promega, Madison, WI, USA) encoding the NF-κB consensus sequence (5′-AGTTGAGGGGACTTTCCCAGGC-3′) and 5′ end-labelled with ©-^32^P (~50,000 cpm) was then added, and the reaction mix incubated for 20 min at room temperature. DNA–protein complexes were resolved on 4 % nondenaturing polyacrylamide gels in 0.5 × TBE (45 mmol/L Tris–borate, 1 mmol/L EDTA, pH 8.0) at 4 °C. Gels were dried and exposed for autoradiography at −80 °C.

### Measurement of the pentose phosphate pathway (PPP) flux

Carbon flux from glucose through the PPP was measured in cells treated for 120 min, as previously described [8], with minor modifications. Briefly, suspensions of known amounts of cells (3–5 × 10^5^ cells) obtained by smooth detaching from the cultures were incubated in sealed vials containing a central well with KOH (4 mol), which was used for ^14^CO_2_ trapping, in the presence of 0.5 μCi of D-[1-^14^C]glucose (activity 56 mCi/mmol/L; Perkin Elmer) or D-[6-^14^C]glucose (activity 60 mCI/mmol/L; Perkin Elmer) in a Krebs–Ringer phosphate buffer (5.7 mmol/L Na_2_HPO_4_, 145 mmol/L NaCl, 4.86 mmol/L KCl, 1.22 mmol/L MgSO_4_, 0.54 mmol/L CaCl_2_; pH 7.4) containing 5.5 or 22 mmol/L glucose at 37 °C. In order to ensure an adequate O_2_ supply for oxidative metabolism by the cells throughout the 120 min incubation period, the gas phase in the vials containing the cells was supplied with extra O_2_ before the vials were sealed. The PPP flux was measured by assessing the difference between ^14^CO_2_ production from [1-^14^C]glucose—which decarboxylates via the 6-phosphogluconate dehydrogenase-catalyzed reaction and the tricarboxylic acid cycle—and that of [6-^14^C]glucose—which decarboxylates only via the tricarboxylic acid cycle [8, 9].

### NADPH oxidase activity

NADPH oxidase activity was measured by lucigenin-derived chemiluminescence, as described previously [10]. After the various treatments described, the cells were washed in ice-cold PBS, scraped and centrifuged at 13,000 rpm for 1 min at 4 °C. The resulting cell pellet was homogenized in lysis buffer (pH 7.0) containing 50 mmol/L KH_2_PO_4_, 1 mmol/L EGTA and 150 mmol/L sucrose for 5 min at 4º C. The activity of NADPH oxidase was measured by lucigenin-derived chemiluminescence. For oxidase assay, cells samples (5 µg protein) were incubated in PBS containing 5 µmol/L lucigenin and 100 µmol/L NADPH. Luminiscence was measured every 30 s for 5 min in a tube luminometer (Optocomp, MGM Instruments, Hamden, CT, USA). The enzymatic activity was obtained as relative light units (RLU)/µg protein min^−1^ determined by the BCA method.

### Determination of total glutathione and GSH

After 24 h of serum deprivation, cells were incubated for a further 18 h period, with the different treatments in serum-free medium. After the treatment described, the cells were washed in ice-cold PBS, scraped and centrifuged at 1200 rpm for 8 min at 4 °C. Total glutathione and GSH were determined in the resulting pellet using a commercial kit (Glutathione Colorimetric Detection kit, Arbor Assays, Ann Arbor, MI, USA).

### siRNA transfection

G6PD silencing was achieved by a procedure previously described [11]. For silencing, the On-TARGETplus SMARTpool for human G6PD siRNA (Dharmacon, Uppsala, Sweden) was used, according to manufacturer’s instructions. The On-Targetplus Non-targeting siRNA #1 (Dharmacon) was used as a negative control. Afterwards, cells were serum-deprived for 24 h and then submitted to the appropriate treatments for Western blot or NADPH oxidase activity determination, as described above.

### Reactivity studies in rat mesenteric microvessels

Isolated mesenteric microvessels from 16-week male old Sprague–Dawley (SD) rats (300–350 g) were studied ex vivo. The animals were briefly exposed to a chamber filled with carbon dioxide until they fell unconscious and then immediately killed by cervical dislocation. The mesentery was removed, and placed in a Petri dish containing Krebs-Henseleit solution (KHS) at 4 °C. The third branch mesenteric arteries were dissected (mean internal diameter ranged between 200 and 400 µm; non-significant differences were observed among the different groups of rats). The arteries were dissected and cleaned free of fat and connective tissue under a light microscope and mounted as ring preparations on a small vessel myograph [12] to measure isometric tension. Arteries were bathed in KHS at 37 °C continuously bubbled with a 95 % O_2_-5 % CO_2_ mixture, which yields a pH of 7.4 and their passive tension and internal circumference were determined. The arteries were subjected to optimal tension (90 % of the tension equivalent to a intramural pressure of 100 mm Hg. After 30 min of equilibration, the vessels were exposed to 125 mmol/L KCL (KKHS, equimolar substitution of KCl for NaCl in KHS) for 2 min in order to check their functional integrity. Segments failing to achieve a maximum active tension equivalent to a pressure of 100 mmHg were rejected [12].

The bath was then washed three times with KHS and a further period of 180 min washout period was allowed with KHS containing 5.5 or 22 mmol/L glucose before the arteries were contracted with the concentration of NA (1 µmol/L) required to produce approximately 80 % of the maximum response to KKHS. Relaxations to acetylcholine (ACh) were subsequently assessed by adding cumulative concentrations of the drug at 2 min intervals (final bath concentrations 0.1 nmol/L–10 µmol/L). Some microvessels were pre-incubated with 2.5 ng/mL IL1β for 120 min before and during the administration of NA and ACh. Other vascular segments were treated with 2 mmol/L 6-aminonicotinamide or 10 µmol/L apocynin for 30 min in advance and during the administration of IL1β, NA, and ACh. The composition of KHS (mmol/L) was NaCl 115, CaCl_2_ 2.5, KCl 4.6, KH_2_PO_4_ 1.2, MgSO_4_.7H_2_O 1.2, NaHCO_3_ 25, glucose 5.5 or 22, and Na_2_EDTA 0.03.

### Materials

Culture plasticware was obtained from TPP (Tragadingen, Switzerland). All the reagents and drugs were purchased from Sigma unless otherwise stated.

### Statistical analysis

Results are expressed as mean ± standard error of at least three separate experiments. Statistical analysis was performed using one-way or two-way ANOVA and Bonferroni correction for multiple comparisons, with the level of significance selected as *P* < 0.05.

## Results

### IL1β enhances glucose transport capacity and consumption

**Fig. 1:**
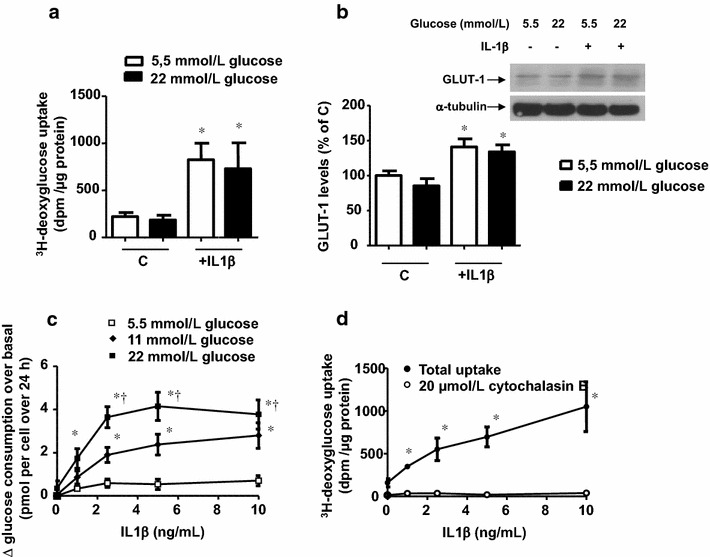
Inflammation, but not exposure to high extracellular glucose, enhances consumption of glucose. **a** Cytochalasine-B sensitive uptake of ^3^H-deoxyglucose by cells previously treated for 18 h with 10 ng/mL IL1β in medium containing 5.5 or 22 mmol/L glucose. Results are the mean ± standard error of 13–15 separate experiments. *P < 0.05 vs the respective control (c). **b** GLUT1 levels, determined by Western blot, in cells exposed for 18 h to IL1β (10 ng/mL) in medium initially containing 5.5 or 22 mmol/L glucose. The gels and blots shown are representative of 4–5 separate experiments. Data are expressed as percentage of the basal expression in cells in medium initially containing 5.5 mmol/L glucose *P < 0.05 vs the respective control (c). **c** Cytokine-induced consumption of glucose in cells exposed for 24 to different concentrations of IL1β (1–10 ng/mL). Data were calculated by subtracting at every point the basal consumption of glucose. Results are expressed as mean ± standard error of 8–11 separate experiments. *P < 0.05 vs 5.5 mmol/L glucose. ^†^ *P* < 0.05 vs 11 mmol/L glucose. **d** Uptake of ^3^H-deoxyglucose by cells previously treated for 18 h with different concentrations of IL1β (1–10 ng/mL) in medium containing 5.5 mmol/L glucose in the presence or absence of the glucose transporter inhibitor cytochalasin B (20 µmol/L). Results are the mean ± standard error of 3 separate experiments. *P < 0.05 vs cytochalasin B-treated cells

At a physiological concentration of 5.5 mmol/L glucose, the cells consumed 2.1 ± 0.3 and 5.3 ± 0.3 pmol per cell during 8 and 24 h, respectively, exhibiting a predominantly glycolytic metabolism, as shown by the lactate: glucose ratio (Table 1a). Increasing the extracellular glucose concentration to 11 or 22 mmol/L affected neither the glucose consumption nor the lactate: glucose ratio (Additional file 1: Figure S1 in the online-only data supplement and Table 1a). Furthermore, it did not increase the total glucose transport capacity (Fig. 1a) nor the expression of GLUT1, the predominant glucose transporter described in vascular cells [13] (Fig. 1b and Additional file 1: Figure S2).

**Table 1:** Glucose consumption and lactate release over 24 h, and ratio between lactate production and glucose consumption in human vascular smooth muscle cells in culture

(a) Control										
	Basal				10 ng/mL IL-1β			0.5 mmol/L sodium azide		
Initial extracellular glucose (mmol/L)	Glucose consumption (pmol per cell)		Lactate production (pmol per cell)	Lactate: glucose ratio	Glucose consumption (pmol per cell)	Lactate production (pmol per cell)	Lactate: glucose ratio	Glucose consumption (pmol per cell)	Lactate production (pmol per cell)	Lactate: glucose ratio
5.5	5.3 ± 0.3	7.0 ± 0.6		1.3 ± 0.1	6.0 ± 0.3*	7.1 ± 0.6	1.2 ± 0.1	6.9 ± 0.1*	8.8 ± 0.9	1.3 ± 0.1
11	4.9 ± 0.6	6.4 ± 1.2		1.3 ± 0.2	8.1 ± 0.9*^†^	9.7 ± 1.7^†^	1.2 ± 0.1			
22	5.5 ± 0.5	7.6 ± 0.8		1.4 ± 0.1	10.0 ± 0.9*^†^	13.0 ± 1.6*^†^	1.3 ± 0.1	13.2 ± 0.8*^†^	17.7 ± 2.2*^†^	1.4 ± 0.2

(b) EV and GLUT1+						
Initial extracellular glucose (mmol/L)	Glucose consumption		Lactate production		Lactate:glucose ratio	
	EV	GLUT1+	EV	GLUT1+	EV	GLUT1+
5.5	6.5 ± 0.7	6.0 ± 0.1	7.9 ± 0.4	6.7 ± 0.5	1.2 ± 0.1	1.1 ± 0.1
22	6.5 ± 0.4	6.2 ± 0.2	9.3 ± 1.0	8.6 ± 0.3	1.5 ± 0.1	1.4 ± 0.2

Results are expressed as the mean ± standard error of 5–28 separate experiments

*EV* cells with a lentiviral empty vector, *GLUT1+* cells with a lentiviral vector containing human GLUT1 cDNA

*P < 0.05 vs the respective control. ^†^ *P* < 0.05 vs the respective value in 5.5 mmol/L glucose

When cells were activated with IL1β (1–10 ng/mL), however, glucose consumption was enhanced in a concentration-dependent manner (Fig. 1c). Moreover, at each concentration of IL1β, the consumption of glucose was proportional to its extracellular concentration (Fig. 1c), although the lactate: glucose ratio was not significantly modified (Table 1a). The increased consumption of glucose caused by IL1β was related to increased GLUT1 mRNA (by 70.4 ± 16.3 %; *P* < 0.05; n = 3) and a higher expression of GLUT1 (Fig. 1b and Additional file 1: Figure S2) that enhanced glucose transport capacity (Fig. 1d). The IL-1 receptor antagonist anakinra (1 µg/mL) abolished the ability of IL1β to enhance the transport capacity of glucose (^3^H-deoxyglucose uptake was 171.6 ± 19.3, 493.3 ± 65.0, and 177.8 ± 4.0 dpm/µg protein in control, IL1β and IL1β plus anakinra, respectively, incubated in 5.5 mmol/L glucose; *P* < 0.05, n = 4). Enhancing glucose concentration, however, did not affect the effects of IL1β on GLUT1 expression or glucose transport capacity (Fig. 1a and 1b, and Additional file 1: Figure S2).

### Glucose overload is not sufficient to cause cell inflammation

**Fig. 2:**
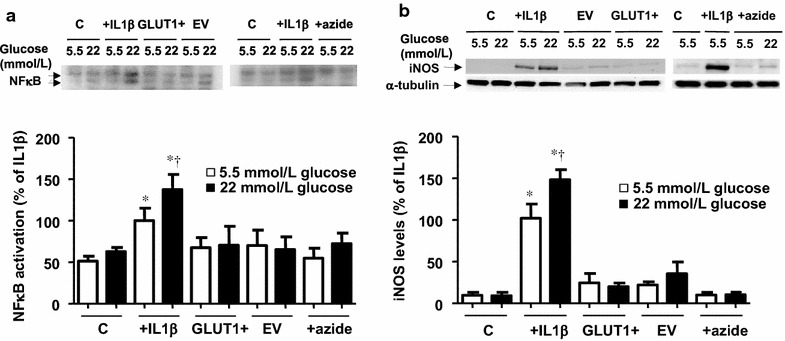
In the absence of IL1β, glucose overload does not evoke pro-inflammatory responses. **a** NF-κB activity, determined by electrophoretic mobility shift assay (EMSA), and **b** iNOS levels, determined by Western blot, in cells exposed to IL1β (10 ng/mL) or sodium azide (0.5 mmol/L), as well as in GLUT1-transfected (GLUT1+) and empty vector (EV)-transfected cells. In each case, the cultures were incubated for 18 h in medium initially containing 5.5 or 22 mmol/L glucose. The gels and blots shown are representative of 4–5 independent experiments. Data are expressed as percentage of the activation or expression produced by IL1β (10 ng/mL) in cells in medium initially containing 5.5 mmol/L glucose *P < 0.05 vs the respective control (c). ^†^ *P* < 0.05 vs respective value in 5.5 mmol/L glucose

We confirmed our previous findings that activation of cells with IL1β resulted in cell inflammation [6], determined as the activation of NFκB and the expression of iNOS. This effect was exaggerated in the presence of high glucose (Fig. 2a, b), through a mechanism independent of hyperosmolarity [6]. Because of the above, we decided to investigate whether the simple increase in the entry of glucose was sufficient to cause inflammation.

In order to achieve this, we first over-expressed GLUT1 transporters by infecting the cells with a lentiviral vector containing human GLUT1 cDNA (GLUT1+). Over-expression of GLUT1 was verified by quantification of GLUT1 mRNA levels (Additional file 1: Figure S3A). While the glucose transport capacity was increased by approximately 50 % in GLUT1+ cells but not in control cells transduced with an empty vector (EV) (Additional file 1: Figure S3B), glucose consumption was not significantly enhanced when the extracellular glucose concentration was increased to 22 mmol/L (Table 1b). Moreover, lactate production and the lactate: glucose ratio in GLUT1+ and EV cells was similar to those in control cells (Table 1b).

In a different set of experiments, we increased glucose entry in the cells by inhibiting mitochondrial respiration using sodium azide (0.5 mM). This treatment did not affect cell survival after 24 h (data not shown), however it increased glucose transport (^3^H-deoxyglucose uptake of 171.6 ± 19.3 and 345.3 ± 46.7 dpm/µg protein in control and plus sodium azide, respectively; *P* < 0.05, n = 4). This was associated with an increase in surface GLUT1 transporters (Additional file 1: Figure S4), without a modification in GLUT1 mRNA levels (98.6 ± 17.0 % vs non-treated cells, n = 3). Sodium azide treatment produced a marked increase in glucose utilization, without changing the lactate: glucose ratio significantly (Table 1a).

Neither the over-expression of GLUT1 nor the glucose overload after sodium azide treatment were sufficient to induce inflammation (Fig. 2a, b).

### IL1β modifies the intracellular metabolic profile of glucose

**Fig. 3:**
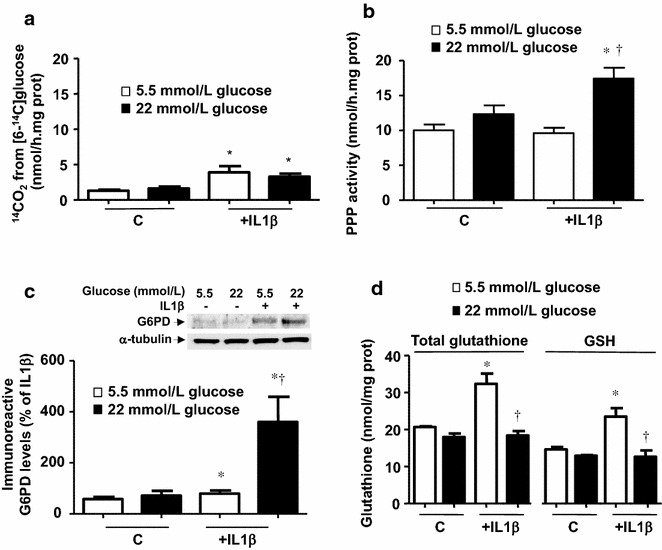
IL1β modifies glucose metabolic profile, activating the pentose phosphate pathway (PPP). (**a**) Cells were treated for 18 h with IL1β (10 ng/mL) in medium containing 5.5 or 22 mmol/L glucose and the ^14^CO_2_ production was measured from [6-^14^C]glucose. Results are the mean ± standard error of 8–12 separate experiments. **b** Cells were treated for 18 h with IL1β (10 ng/mL), in medium containing 5.5 or 22 mmol/L glucose. The PPP flux was then measured for 120 min by assessing the difference between ^14^CO_2_ production from [1-^14^C]glucose—and that of [6-^14^C]glucose. Results are the mean ± standard error of 8–12 separate experiments. (**c**) G6PD levels, determined by Western blot, in cells treated for 18 h with IL1β (10 ng/mL), in medium containing 5.5 or 22 mmol/L glucose. Results are the mean ± standard error of 5 separate experiments, expressed as percentage of the expression produced by treatment with IL1β in medium containing 5.5 mmol/L glucose. (**d**) Total glutathione and GSH production in cells exposed for 18 h to IL1β (10 ng/mL) in medium containing 5.5 or 22 mmol/L glucose. Results are the mean ± standard error of 3–5 separate experiments. *P < 0.05 vs respective control (c). ^†^ *P* < 0.05 vs respective value in 5.5 mmol/L glucose

We next monitored the intracellular utilization of glucose by measuring the generation of ^14^CO_2_ from glucose labelled in different carbon atoms. The production of ^14^CO_2_ from [6-^14^C]glucose, which decarboxylates only via the tricarboxylic acid cycle, was not modified by incubation in a high concentration of glucose (Fig. 3a). There was, however, a significant increase following treatment with IL1β, which was similar at both 5.5 and 22 mmol/L glucose (Fig. 3a). The activity of the pentose phosphate pathway (PPP) was not significantly affected by increasing the concentration of glucose in the medium or by incubating cells in 5.5 mmol/L glucose with IL1β (10 ng/ml); however, a significant enhancement was observed when the cells were activated by IL1β in a medium containing 22 mmol/L glucose (Fig. 3b). The protein levels of glucose-6-phosphate dehydrogenase (G6PD), as the step-limiting enzyme of the PPP, were slightly increased after IL1β treatment in 5.5 mmol/L glucose, but markedly enhanced in 22 mmol/L glucose (Fig. 3c).

### Over-activation of NADPH oxidase by IL1β and high glucose

NADPH generated by the PPP can be used either to regenerate reduced glutathione (GSH) through the action of glutathione reductase or as a substrate to fuel NADPH oxidase. We found that incubation in 5.5 mmol/L glucose with IL1β increased both total glutathione and GSH; however, this effect was abolished in cells incubated in 22 mmol/L glucose (Fig. 3d). In the absence of the cytokine, simply enhancing the extracellular concentration of glucose failed to modify the intracellular glutathione profile (Fig. 3d).

**Fig. 4:**
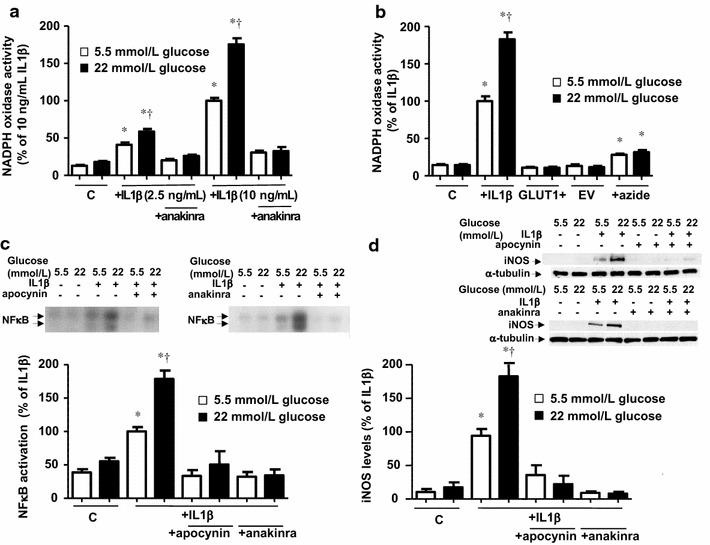
High glucose medium potentiates the NADPH oxidase activation induced by IL1β. (**a**) Cells were exposed to IL1β (2.5 and 10 ng/mL), in the presence or absence of the IL1 receptor antagonist anakinra (1 µg/mL), in medium initially containing 5.5 or 22 mmol/L glucose. NADPH oxidase activity was determined by lucigenin-derived chemiluminescence after 18 h. Results are the mean ± standard error of 3–15 separate experiments expressed as percentage of the relative light units produced by 10 ng/mL IL1β in medium initially containing 5.5 mmol/L glucose (472.7 ± 30.3 RLU/µg protein min^−1^). (**b**) NADPH oxidase activity in cells exposed to IL1β (10 ng/mL) or sodium azide (0.5 mmol/L) and in GLUT1- and EV-transfected cells after 18 h of incubation in medium initially containing 5.5 or 22 mmol/L glucose. Results are the mean ± standard error of 3–5 separate experiments expressed as percentage of the relative light units produced by 10 ng/mL IL1β in medium initially containing 5.5 mmol/L glucose (457.1 ± 64.0 RLU/µg protein min^−1^). (**c**) NFκB activity, measured by EMSA, and (**d**) iNOS levels, determined by Western blot, in cells treated for 18 h with IL1β (10 ng/mL), in the presence or absence of the NADPH oxidase inhibitor apocynin (30 µmol/L) or anakinra (1 µg/mL), in medium containing 5.5 or 22 mmol/L glucose. Results are the mean ± standard error of 3–4 separate experiments expressed as percentage of the activation or expression produced by 10 ng/mL IL1β in medium initially containing 5.5 mmol/L glucose. *P < 0.05 vs respective control (c). ^†^ *P* < 0.05 vs respective value in 5.5 mmol/L glucose

Measurements of the NADPH oxidase showed that IL1β elicited a concentration-dependent activation of this enzyme, which was exaggerated in the presence of high glucose (Fig. 4a). NADPH oxidase activity was not modified by just enhancing glucose transport capacity or following mitochondrial inhibition (Fig. 4b). By using the antioxidant and NADPH oxidase inhibitor apocynin as well as anakinra, we observed that the activation of NADPH oxidase via IL-1 receptors was necessary to induce inflammation by IL1β and its enhancement by high glucose (Figs. 4c, d).

### The over-activation of NADPH oxidase depends on the PPP activation

**Fig. 5:**
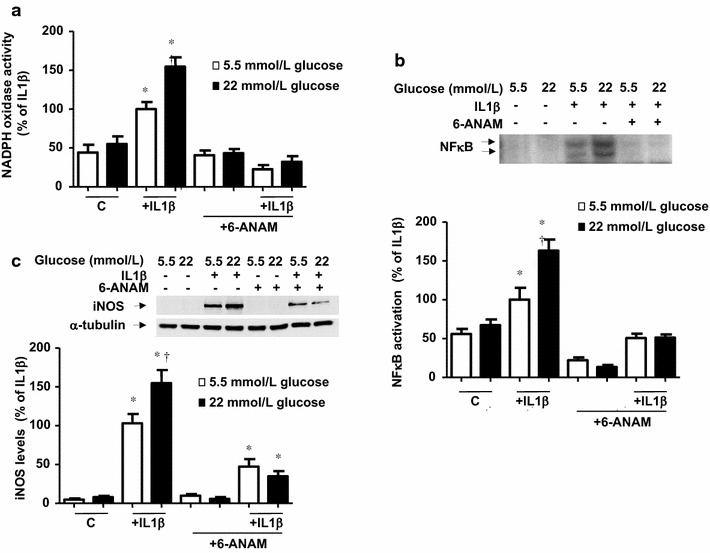
Pharmacological blockade of G6PD abrogates the pro-inflammatory action by IL1β and its potentiation by glucose. (**a**) NADPH oxidase activity, determined by lucigenin-derived chemiluminescence in cells exposed to IL1β (10 ng/mL) after 18 h of incubation, in the presence or absence of 6-aminonicotinamide (6-ANAM; 2 mmol/L), in medium initially containing 5.5 or 22 mmol/L glucose. Results are the mean ± standard error of 3–6 separate experiments expressed as percentage of the relative light units produced by 10 ng/mL IL1β in medium initially containing 5.5 mmol/L glucose (380.1 ± 59.7 RLU/µg protein min^−1^). (**b**) NFκB activation, measured by EMSA, and (**c**) iNOS levels, determined by Western blot, after 18 h exposure of cells to IL1β (10 ng/mL), in the presence or absence of 6-ANAM (2 mmol/L) in medium containing 5.5 or 22 mmol/L glucose. Results are the mean ± standard error of 3–5 separate experiments expressed as percentage of the activation or the expression produced by treatment with IL1β in medium containing 5.5 mmol/L glucose. *P < 0.05 vs respective control (c). ^†^ *P* < 0.05 vs respective value in 5.5 mmol/L glucose

**Fig. 6:**
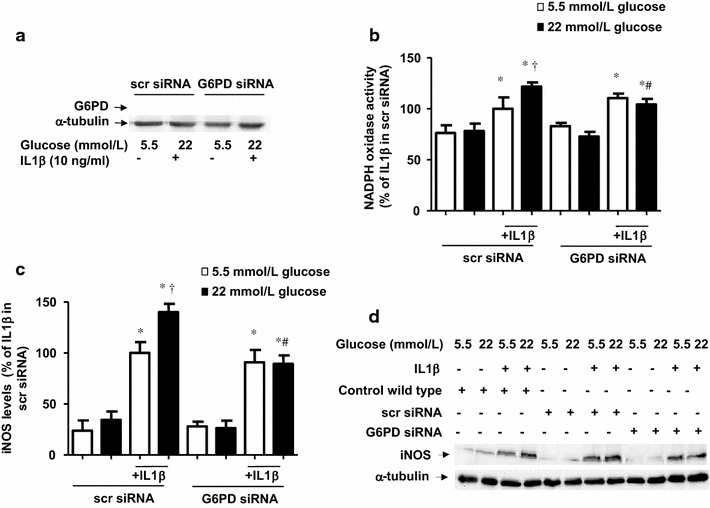
G6PD siRNA abrogates the glucose potentiation of IL1β-evoked pro-inflammatory response. (**a**) G6PD levels, determined by Western blot, in cells treated with scrambled siRNA or G6PD siRNA and submitted for 18 h IL1β (10 ng/mL) in medium containing 5.5 or 22 mmol/L glucose. (**b**) NADPH oxidase activity, determined by lucigenin-derived chemiluminescence in cells treated with scrambled siRNA and G6PD siRNA and exposed to IL1β (10 ng/mL) during 18 h of incubation, in medium initially containing 5.5 or 22 mmol/L glucose. Results are the mean ± standard error of 3–6 separate experiments expressed as percentage of the relative light units produced by 10 ng/mL IL1β in control cells incubated in a medium initially containing 5.5 mmol/L glucose (380.1 ± 59.7 RLU/µg protein min^−1^). (**c** and **d**) iNOS levels, determined by Western blot, in cells untreated or treated with scrambled (scr)-siRNA and G6PD siRNA and exposed to IL1β (10 ng/mL) during 18 h of incubation in medium initially containing 5.5 or 22 mmol/L glucose. The gels and blots are representative of 3–5 separate experiments, while the bars are expressed as percentage of the activation or the expression produced by treatment with IL1β in sc-siRNA cells incubated in a medium with 5.5 mmol/L glucose. *P < 0.05 vs respective control (c). ^†^ *P* < 0.05 vs respective value in 5.5 mmol/L glucose. ^#^ *P* < 0.05 vs respective value in scr-siRNA

To study the role of the PPP on the activation of NADPH oxidase, we used the G6PD pharmacological inhibitor 6-aminonicotinamide (6-ANAM). In cells activated with IL1β this compound prevented the activation of NADPH oxidase and the resulting inflammation at 5.5 and 22 mmol/L glucose (Figs. 5a, b, c). To obtain a more selective blockade of G6PD activity, cells were treated with G6PD siRNA (Fig. 6a). There was a significant increase (*P* < 0.005) in the basal NADPH oxidase activity and iNOS expression produced both by scrambled or G6PD siRNA, suggesting the experimental procedure may originate some unspecific inflammation. Interestingly, however, G6PD silencing specifically blocked the over-activation of NADPH oxidase and the enhanced iNOS expression induced by high glucose (Figs. 6b, c, d).

### High glucose potentiates endothelial dysfunction induced by IL1β by a mechanism involving NADPH oxidase and PPP

**Fig. 7:**
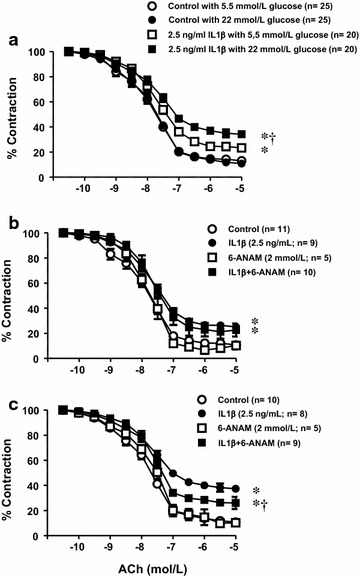
Endothelial dysfunction evoked by IL1β in rat mesenteric microvessels. (**a**) Effect of IL1β (2.5 ng/mL) on the endothelium-dependent relaxations induced by acetylcholine (ACh, 0.1 nmol/L to 10 µmol/L) in isolated mesenteric arteries from Sprague–Dawley (SD) rats incubated with medium containing 5.5 or 22 mmol/L glucose. Data are expressed (mean ± SE) as the percentage of the previous contraction induced with 1 µmol/L noradrenaline (NA), which averaged 9.36 ± 0.77, 9.33 ± 0.58, 9.04 ± 0.83, and 9.67 ± 0.74 mNewtons, respectively. The number of segments used for every curve, which were obtained from 12 animals are in parenthesis. *P < 0.05 vs respective control. ^†^ *P* < 0.05 vs respective value in 5.5 mmol/L glucose. (**b**) Effect of 6-ANAM (2 mmol/L) on the endothelium impairment induced by IL1β in rat vascular mesenteric segments incubated in 5.5 mmol/L glucose. Data are expressed (mean ± SE) as the percentage of the previous contraction induced with NA, which averaged 10.67 ± 0.76, 10.10 ± 0.84, 12.10 ± 1.37, and 10.83 ± 1.14 mNewtons, respectively. The number of segments used for every curve, which were obtained from 5 animals are in parenthesis. *P < 0.05 vs control. (**c**) Effect of 6-ANAM on the endothelium impairment induced by IL1β in rat vascular mesenteric segments incubated in 22 mmol/L glucose. Data are expressed (mean ± SE) as the percentage of the previous contraction induced with NA, which averaged 10.07 ± 0.55, 10.30 ± 0.71, 9.27 ± 1.76, and 10.87 ± 1.15 mNewtons, respectively. The number of segments used for every curve, which were obtained from 5 animals are in parenthesis. *P < 0.05 vs control. ^†^ *P* < 0.05 vs IL1β

We first confirmed our previous results [12] demonstrating that treating isolated segments of rat mesenteric microvessels with 2.5 ng/ml produced an impairment of the endothelium-dependent relaxations evoked by ACh through a mechanism dependent on NADPH oxidase (Fig. 7a; Additional file 1: Figure S5). Moreover, we observed that the endothelial dysfunction induced by the cytokine was further enhanced when the vascular segments were pre-incubated in a medium with 22 mmol/L glucose (Fig. 7a). The role of the PPP was studied by treating the mesenteric microvessels with 6-ANAM; this drug did not affect the endothelial dysfunction induced by IL1β in normal glucose but prevented the potentiation observed in high glucose conditions (Figs. 7b, c).

## Discussion

Our results clearly show that the mere elevation of extracellular glucose does not result in increased uptake by human vascular smooth muscle. Indeed, non-inflamed cells exhibit a glycolytic profile, which is in line with previous findings [14, 15], with no changes observed in glucose consumption and lactate generation when the extracellular glucose was elevated. The lack of increase in glucose consumption with increasing concentrations of glucose can be explained by the saturation of its transport; the glucose transporter GLUT1, predominant in vascular cells, has a high affinity and low capacity (Km of 1–7 mmol/L), operating near its maximal capacity at physiological concentrations of glucose [13]. In fact, it has been shown before that enhancing the extracellular concentration of glucose may even down-regulate GLUT1 activity [16].

However, treating these cells with IL1β increased the glucose transport in a concentration-dependent manner. This enhanced glucose uptake was associated with newly synthesized GLUT1 and increased number of transporters in the cell membrane, similar to what occurs in immune cells after inflammatory activation [17]. There was, in addition, an increase in both the consumption of glucose and the generation of lactate, which was proportional to the concentration of glucose in the extracellular medium. Thus, our results also indicate that the cytokine is required to achieve extra glucose entry and consumption by the cells when the extracellular concentrations of glucose are high.

We confirmed that inflammation induced by IL1β was exacerbated in the presence of high concentrations of glucose [6]; then, we decided to investigate whether increasing intracellular glucose was sufficient to induce inflammation. We found that simply over-expressing functional GLUT1 did not lead to high glucose consumption or inflammation. This is in contrast to what has been shown by others in non-vascular cells with a very higher over-expression of GLUT1 [18–20]. Interestingly, however, in animals genetically modified to over-express GLUT1 in vascular smooth muscle inflammation was only observed when vascular damage was superimposed [21]. In addition, inhibition of mitochondrial respiration by sodium azide resulted in enhanced glucose consumption and lactate production to an even greater degree than that observed following activation with IL1β. Inhibition of mitochondrial respiration is known to increase glucose transport through the translocation of pre-existing GLUT1 to the cell membrane [22]). In spite of this, no evidence of glucose-dependent inflammation was observed. Therefore, the enhancement of glucose consumption and glycolytic metabolism is not sufficient to trigger inflammation in vascular cells.

We then investigated whether the stimulation with IL1β modified the glucose metabolism and found that the glucose metabolized via the tricarboxylic acid cycle was only slightly enhanced by the cytokine treatment, independently of the extracellular glucose concentration. This further argues against the hypothesis which suggests a main role for mitochondria-derived superoxide anions in the vascular damage induced by glucose [1]. In fact, the major finding of the present study is the demonstration that the PPP is the pathway through which high glucose exacerbates inflammation in vascular cells. Specifically, high glucose concentrations lead to higher expression of G6PD and augmented PPP activity in cells activated with IL1β, indicating that the cytokine was required for the diversion of the excess intracellular glucose through this metabolic pathway. Previous results in vascular cells suggest that the PPP activity, which is low in basal conditions, can be activated during inflammation [15, 23, 24].

The PPP is a main source for NADPH, which can be used as a cofactor of glutathione reductase for the regeneration of GSH or as a substrate for NADPH oxidase for the release of free radicals. This pro-oxidant enzyme has been suggested to play a key role in diabetes-associated atherosclerosis [25]. In our cells, treatment with IL1β activated NADPH oxidase, which was necessary for the induction of inflammation via the production of reactive oxygen species. In parallel, IL1β increased GSH content, probably as a compensatory mechanism against enhanced free radical formation in inflammation [26]. In the presence of high glucose and PPP over-activation, however, NADPH oxidase activity by IL1β was further increased while the protective effect of GSH was diminished. NADPH oxidase requires higher concentrations of NADPH to be active, as the Michaelis constant for this enzyme is 5-fold higher than for glutathione reductase [27]. Thus, the over-activation of the PPP would favor the utilization of NADPH by NADPH oxidase and the excess of free radical generation would further contribute to exhausting GSH. Furthermore, high glucose has been suggested to decrease the de novo synthesis of glutathione through the down-regulation of the expression of the glutamate-cysteine ligase [26, 28].

Additional evidence supporting the interaction between high glucose and IL1β-induced vascular damage, was provided by functional reactivity experiments in rat mesenteric microvessels demonstrating that a high glucose medium also enhanced the impairment of endothelium-dependent relaxations evoked the cytokine. Moreover, this exacerbated endothelial dysfunction was prevented both by NADPH oxidase inhibition or PPP blockade. As we have previous data demonstrating that the endothelial dysfunction induced by IL1β is due to an increased oxidative stress produced by enhanced NADPH oxidase activity, which is also occurring in diabetes [12], it seems reasonable to conclude that a pathway linking the over-activation of PPP with the NADPH oxidase activity may have a role in several vascular alterations associated to diabetes mellitus.

Although other studies have reported in cardiovascular cells a link between the PPP and NADPH oxidase activation in the context of hyperglycemia and diabetes [29–33], our data clearly single out the role of this pathway as responsible for the exacerbated inflammation and vascular dysfunction induced by high glucose. Thus, inhibition of G6PD in vascular cells abrogated not only the over-activation of NADPH oxidase but also the subsequent exacerbated inflammation and endothelial dysfunction, which may help to explain a number of other experimental and clinical observations. It has been known for many years that diabetic patients have a decreased tissue concentration of GSH [34, 35], while defects in GSH-dependent antioxidant enzymatic activity have been related to diabetes-associated atherosclerosis [36]. Moreover, a lower susceptibility to cardiovascular disease has been described in patients with G6PD deficiency [37, 38], while mice with genetic deficiency in G6PD are protected against atherosclerosis [39]. From a translational approach, it is likely that anti-inflammatory treatment, as adjunct to glucose control, will prove of benefit for the prevention or treatment of cardiovascular complications in diabetes. This has been suggested by the recent use of the IL-1 receptor antagonist anakinra in an animal model of diabetes [12] as well as by studies in patients with anakinra or canakinumab [41].

## Conclusions

**Fig. 8:**
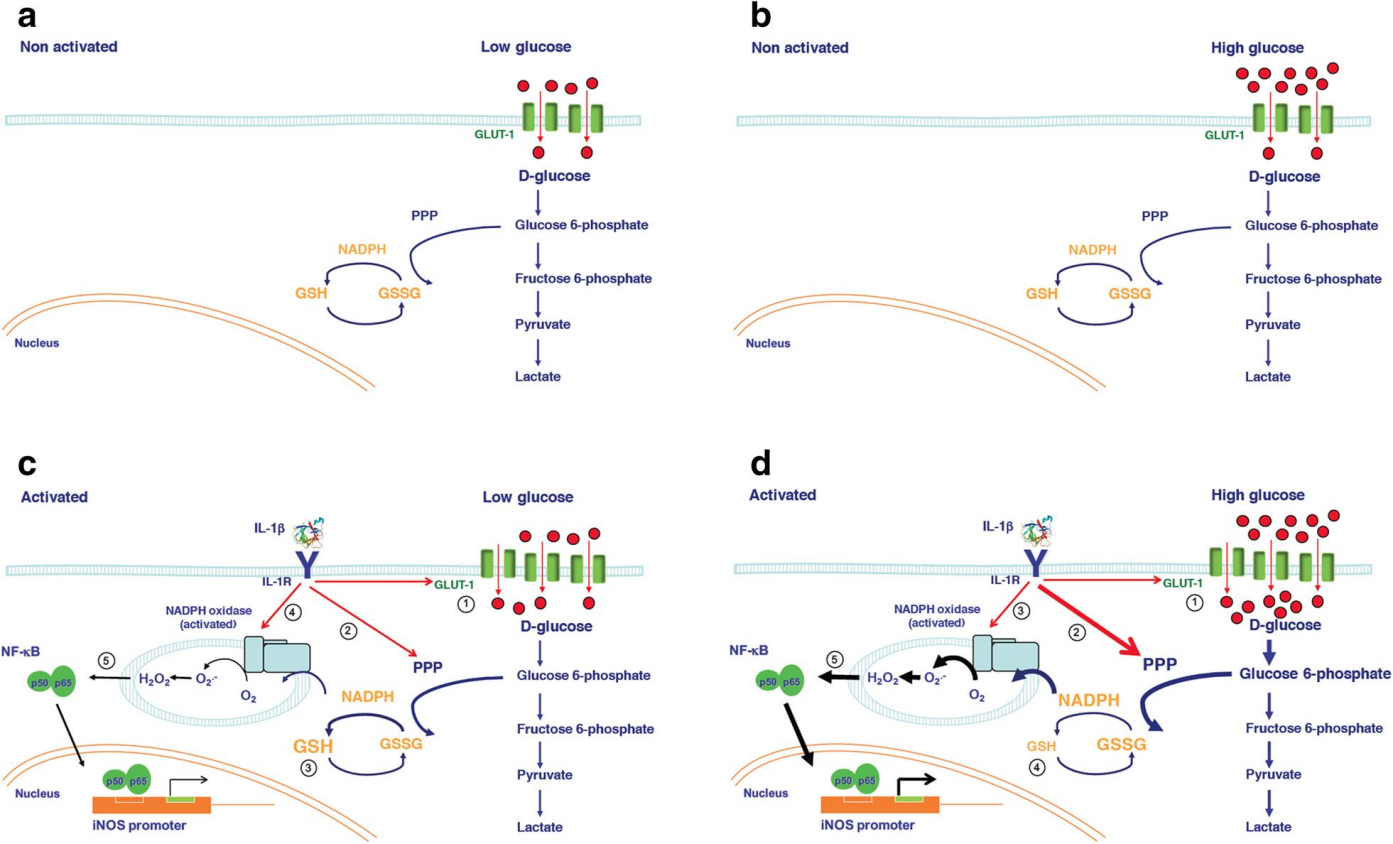
Effects of high glucose on inflamed human vascular smooth muscle cells. (**a** and **b**) Under low or high extracellular glucose and in the absence of cytokine activation, the cell regulates the entry of glucose. (**c**) However, activation with IL1β augments GLUT-dependent glucose uptake ①, enhances PPP activity ② and GSH levels ③, and activates NADPH oxidase ④ and pro-inflammatory signalling ⑤. (**d**) When the cytokine activation coexists with high concentrations of extracellular glucose, the increased glucose entering the cell ① is partially diverted through an over-stimulated PPP ②, resulting in a pro-oxidant environment ③ that cannot be counterbalanced by a reduced regeneration of GSH ④, leading to enhanced inflammation and ultimately to tissue damage ⑤

Our results indicate that over-activation of the PPP is a crucial mechanism by which high glucose exacerbates vascular cell damage. Activation of the PPP by pro-inflammatory cytokines allows excess glucose to enter this metabolic route creating a situation in which free radical formation exceeds the capacity of the cell to regenerate GSH. This pro-oxidant environment increases vascular inflammation and as result, induced the vascular damage associated to hyperglycaemia (Fig. 8). Furthermore, from a therapeutic point of view, our results indicate the necessity not only to control glycaemia but also to reduce inflammation in order to prevent the potential harmful effect of high glucose in vascular cells.

## Additional file

10.1186/s12933-016-0397-2 Supplementary figures.

## Abbreviations

ACh: acetylcholine
6-ANAM: 6-aminonicotinamide
ANOVA: analysis of variance
cDNA: complementary deoxyribonucleic acid
EMSA: electrophoretic mobility shift assay
EV: cells with empty vector
GLUT1: glucose transporter 1
GLUT1+: cells with a lentiviral vector containing human GLUT1 cDNA
G6PD: glucose-6-phosphate dehydrogenase
GSH: reduced glutathione
HASMC: human aortic smooth muscle cells
iNOS: inducible nitric oxide synthase
IL1β: interleukin-1β
mRNA: messenger RNA
NA: noradrenaline
NADPH: nicotine adenine dinucleotide phosphate
NFκB: nuclear factor κB
PPP: pentose phosphate pathway
RT-PCR: reverse transcription polymerase chain reaction
TNF-α: tumour necrosis factor-α
