# Erratum to: Inflammation, glucose, and vascular cell damage: the role of the pentose phosphate pathway

**DOI:** 10.1186/s12933-017-0502-1

**Published:** 2017-02-16

**Authors:** Concepción Peiró, Tania Romacho, Verónica Azcutia, Laura Villalobos, Emilio Fernández, Juan P. Bolaños, Salvador Moncada, Carlos F. Sánchez-Ferrer

**Affiliations:** 1 Departamento de Farmacología, Facultad de Medicina, Universidad Autónoma de Madrid, 29029 Madrid, Spain; 2 Instituto de Biología Funcional y Genómica, Universidad de Salamanca-CSIC, 37007 Salamanca, Spain; 3 Wolfson Institute for Biomedical Research, University College London, London, WC1E 6BT UK; 4 Paul Langerhans-Group, Integrative Physiology, German Diabetes Center, Auf’m Hennekamp 65, 40225 Düsseldorf, Germany; 5 Department of Pathology, University of Michigan, Ann Arbor, MI 48109 USA; 6 Institute of Cancer Sciences, Manchester Cancer Research Centre, University of Manchester, Wilmslow Road, Manchester, M20 4QL UK

## Erratum to: Cardiovasc Diabetol (2016) 15:82 DOI 10.1186/s12933-016-0397-2

After publication of the original article [1], it became apparent that an error affecting Fig. 6 occurred during production. In the published article, Fig. 6a is missing the Western blot corresponding to G6PD.

**Fig. 6:**
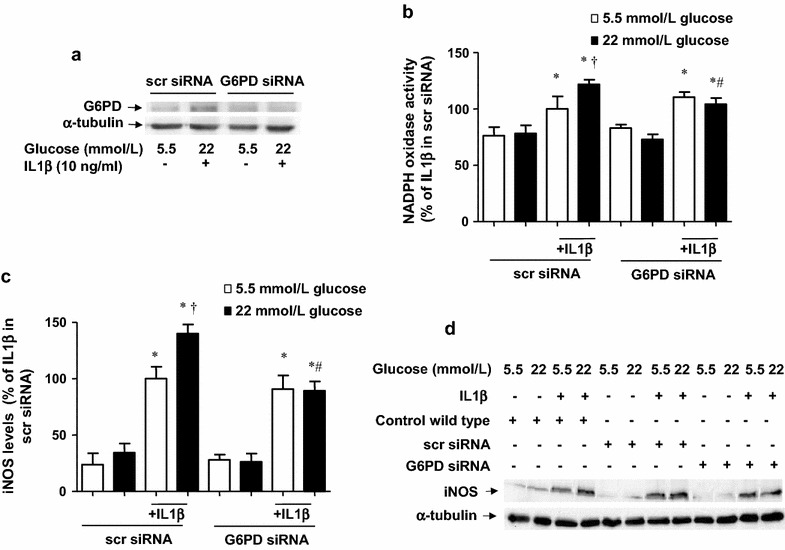
G6PD siRNA abrogates the glucose potentiation of IL1β-evoked pro-inflammatory response. **a** G6PD levels, determined by Western blot, in cells treated with scrambled siRNA or G6PD siRNA and submitted for 18 h IL1β (10 ng/mL) in medium containing 5.5 or 22 mmol/L glucose. **b** NADPH oxidase activity, determined by lucigenin-derived chemiluminescence in cells treated with scrambled siRNA and G6PD siRNA and exposed to IL1β (10 ng/mL) during 18 h of incubation, in medium initially containing 5.5 or 22 mmol/L glucose. Results are the mean ± standard error of 3–6 separate experiments expressed as percentage of the relative light units produced by 10 ng/mL IL1β in control cells incubated in a medium initially containing 5.5 mmol/L glucose (380.1 ± 59.7 RLU/µg protein min^−1^). **c**, **d** iNOS levels, determined by Western blot, in cells untreated or treated with scrambled (scr)-siRNA and G6PD siRNA and exposed to IL1β (10 ng/mL) during 18 h of incubation in medium initially containing 5.5 or 22 mmol/L glucose. The gels and blots are representative of 3–5 separate experiments, while the bars are expressed as percentage of the activation or the expression produced by treatment with IL1β in sc-siRNA cells incubated in a medium with 5.5 mmol/L glucose. *P < 0.05 vs respective control (**c**). ^†^ *P* < 0.05 vs respective value in 5.5 mmol/L glucose. ^#^ *P* < 0.05 vs respective value in scr-siRNA

The correct version of the figure was submitted by the author, and the error occurred during the typesetting stage. The correct version (Fig. 6) of the figure is published in this erratum.
