# Supplementary material for: Inflammation, glucose, and vascular cell damage: the role of the pentose phosphate pathway
Source: Cardiovasc Diabetol. 2016 Jun 1;15:82. doi: 10.1186/s12933-016-0397-2 (PMC4888494; doi:10.1186/s12933-016-0397-2)
Supplement: Supplementary file 1 — 10.1186/s12933-016-0397-2 Supplementary figures. [file 12933_2016_397_MOESM1_ESM.docx]

**Supplementary Figures**

**5.5 mmol/L glucose**

**11 mmol/L glucose**

**22 mmol/L glucose**

**Glucose consumption**

**(pmol per cell)**

**0**

**8**

**24**

**0**

**2**

**4**

**6**

**Time (h)**

**Figure S1.** Basal consumption of glucose by human aortic smooth muscle cells cultured for 8 and 24 h in medium initially containing 5.5, 11, or 22 mmol/L glucose. Results are the mean ± standard error of 13-25 separate experiments.


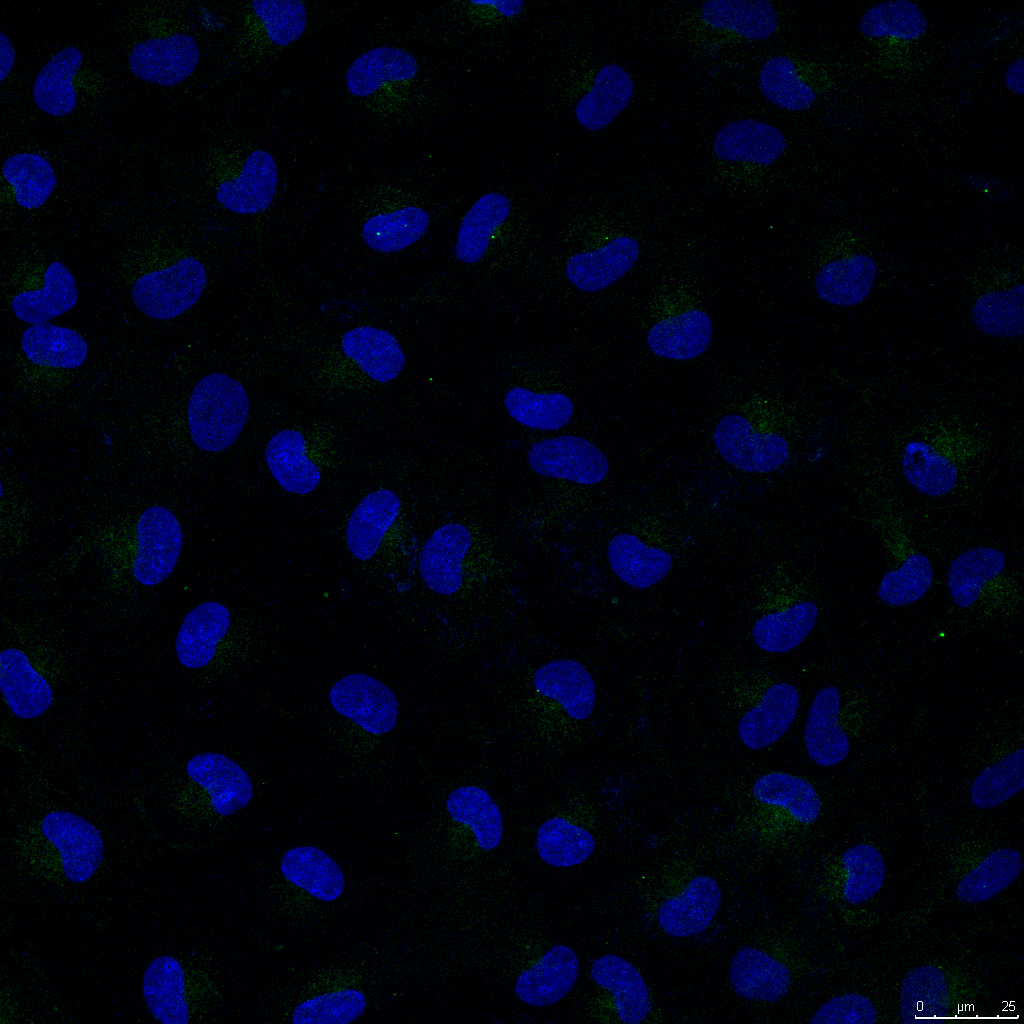

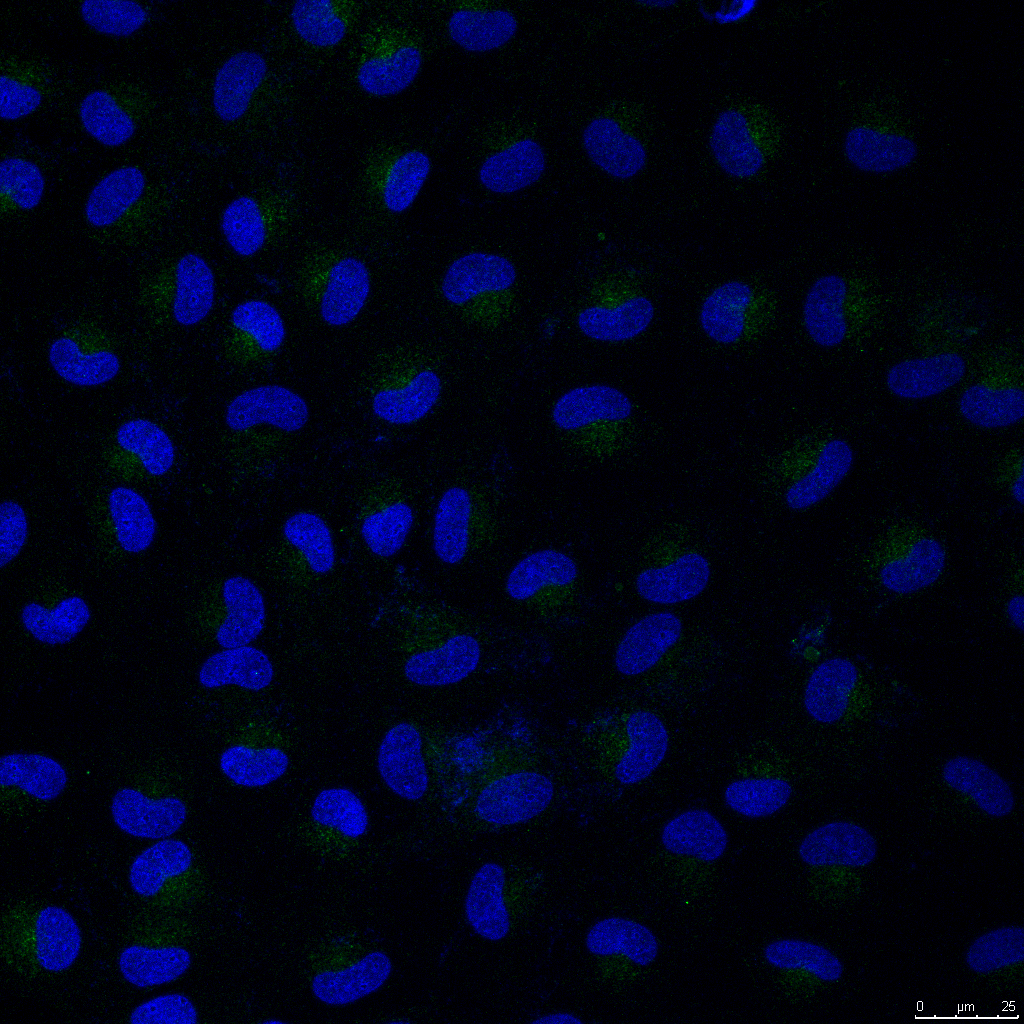

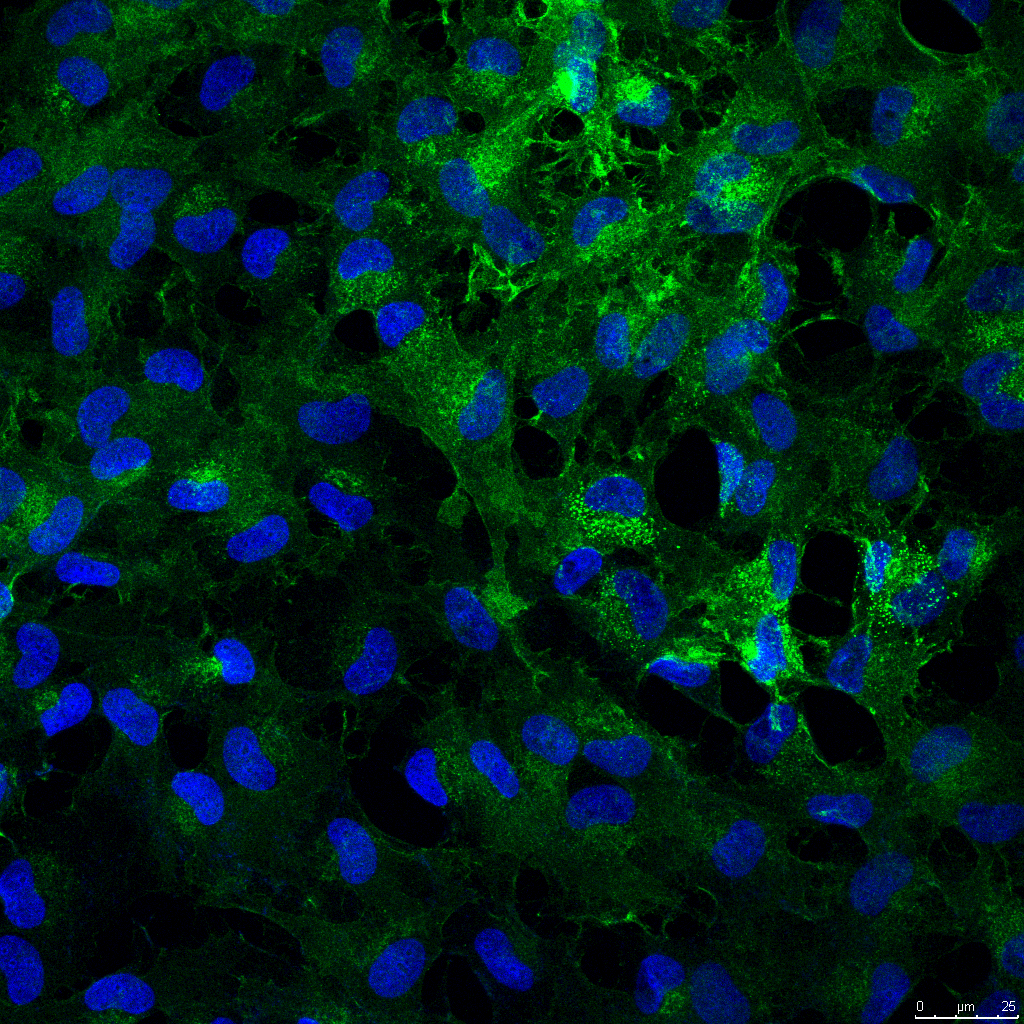

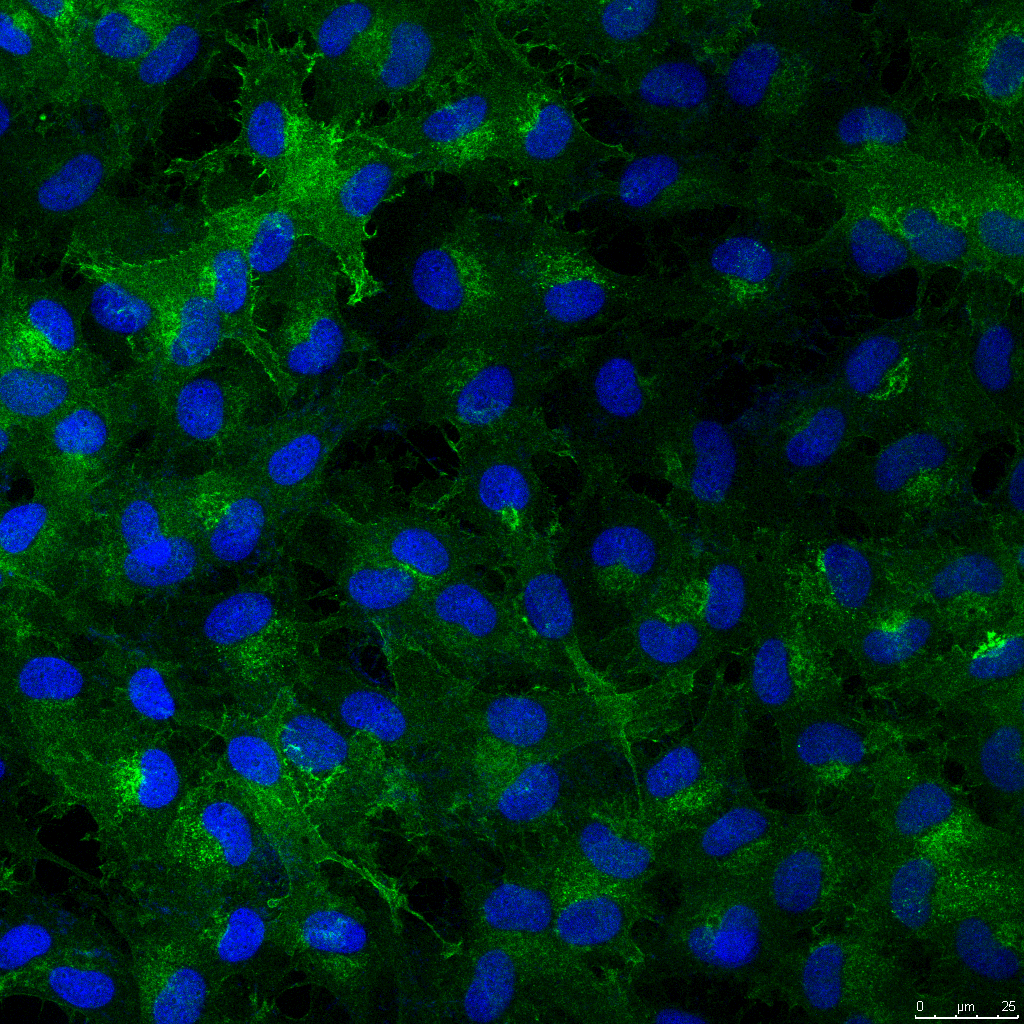


**5,5 mmol/L glucose**

**22 mmol/L glucose**

**5,5 mmol/L glucose+IL1β**

**22 mmol/L glucose+IL1β**

**Figure S2.** Immunofluorescence for GLUT1 transporters (green) in cells exposed for 18 h to IL1β (10 ng/mL) in medium containing 5.5 or 22 mmol/L glucose. Nuclei were counterstained with DAPI (blue).

**
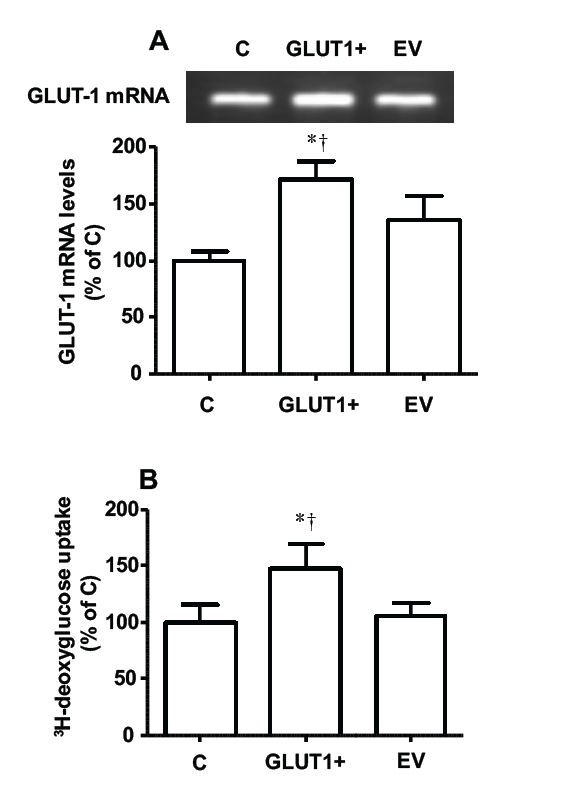
**

**Figure S3. (A)** Representative gel and quantification of GLUT1 mRNA levels determined by PCR in control (C) cells, cells transfected with cDNA for GLUT1 (GLUT1+) and cells infected with an empty vector (EV). Results are expressed as mean±SEM of 3 separate experiments expressed as percentage of the level in control (c) cells. *P<0.05 vs C. †*P*<0.05 vs EV. **(B)** Cytochalasin B-sensitive uptake of ^3^H-deoxyglucose in control, GLUT1+ and EV-transfected cells. Results are mean ± standard error of 14 separate experiments expressed as percentage of the control radioactivity uptake (87.7±14.2 dpm/µg protein). **P*<0.05 vs C. †*P*<0.05 vs EV.

**Control**

**+azide**


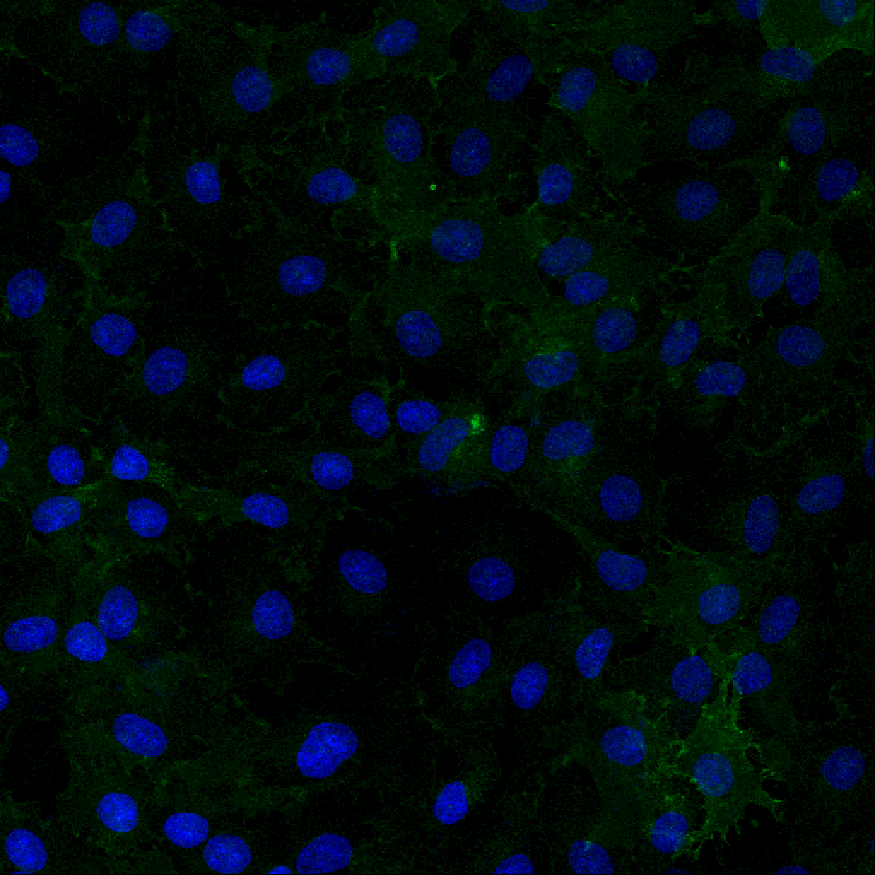

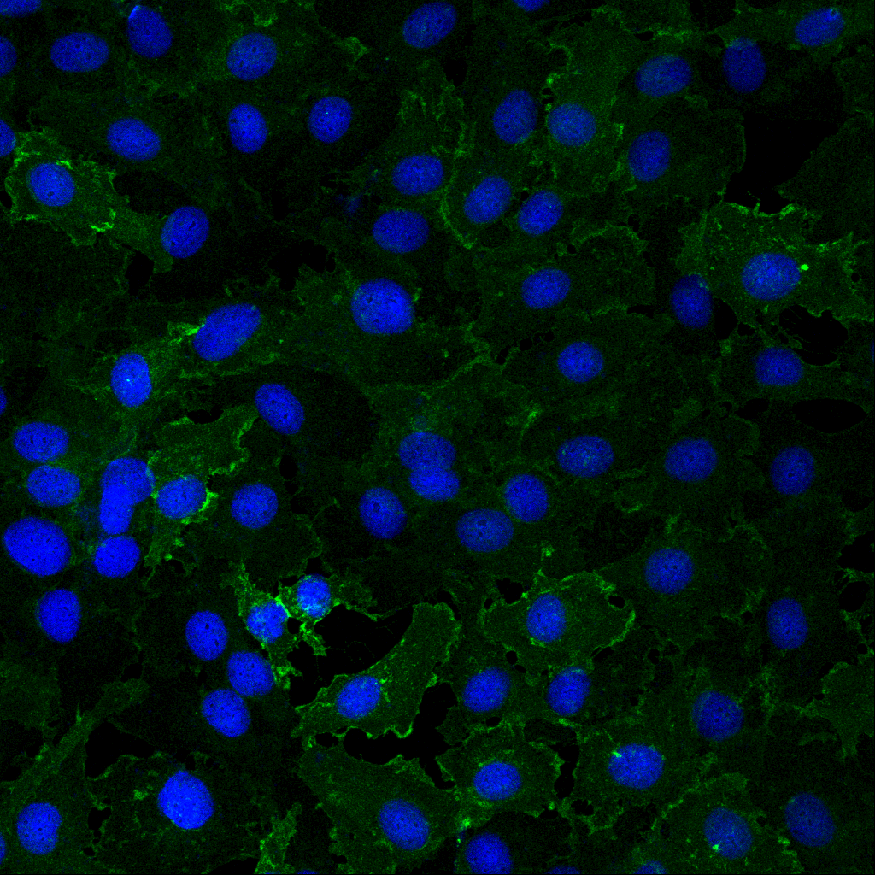


**Figure S4**. Immunofluorescence for GLUT1 transporters (green) in cells exposed for 18 hr to sodium azide (0.5 mmol/L) in medium containing 5.5 mmol/L glucose. Nuclei were counterstained with DAPI (blue).

**
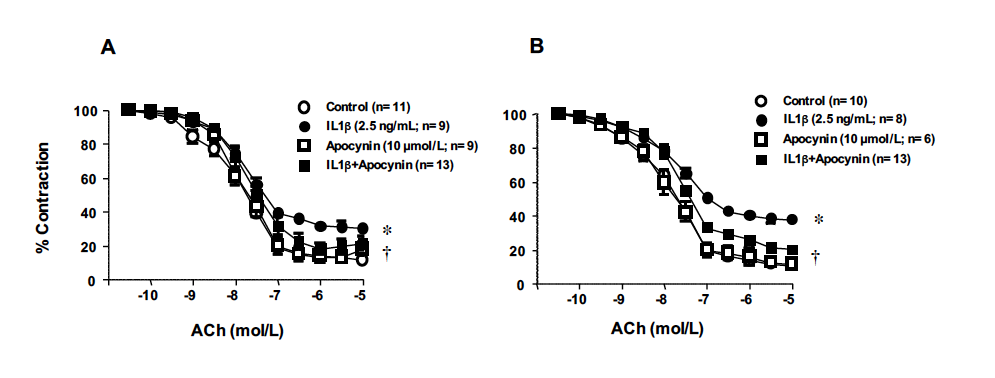
**

**Figure S5.** (**A**) Effect of apocynin (10 µmol/L) on the impairment of the ACh-induced vasodilatations induced by 2.5 ng/ml IL1β in rat vascular mesenteric segments incubated in 5.5 mmol/L glucose. Data are expressed (means ± SE) as the percentage of the previous contraction induced with NA, which averaged 10.78±0.70, 9.61±0.72, 10.39±1.22, and 9.44±1.15 mNewtons, respectively. The number of segments used for every curve, which were obtained from 5 animals, are in parenthesis. **P*<0.05 vs control. (**B**) Effect of apocynin on the endothelium impairment induced by IL1β in rat vascular mesenteric segments incubated in 22 mmol/L glucose. Data are expressed (means ± SE) as the percentage of the previous contraction induced with NA, which averaged 9.87±0.56, 9.88±0.70, 9.16±0.48, and 10.38±1.13 mNewtons, respectively. The number of segments used for every curve, which were obtained from 5 animals are in parenthesis. **P*<0.05 vs control. †*P*<0.05 vs IL1β.
